# Pathogenesis of Preeclampsia and Therapeutic Approaches Targeting the Placenta

**DOI:** 10.3390/biom10060953

**Published:** 2020-06-24

**Authors:** Manoj Kumar Jena, Neeta Raj Sharma, Matthew Petitt, Devika Maulik, Nihar Ranjan Nayak

**Affiliations:** 1 Department of Biotechnology, School of Bioengineering and Biosciences, Lovely Professional University (LPU), Phagwara, Punjab 144411, India; neeta.raj@lpu.co.in; 2 Redwood Biomedical Editing, Redwood City, CA 94061, USA; petitt@mac.com; 3 Department of Obstetrics and Gynecology, UMKC School of Medicine, Kansas City, MO 64108, USA; devika.maulik@gmail.com (D.M.); nrn2kt@umkc.edu (N.R.N.)

**Keywords:** preeclampsia, pathogenesis, cytotrophoblasts, placenta, spiral artery

## Abstract

Preeclampsia (PE) is a serious pregnancy complication, affecting about 5–7% of pregnancies worldwide and is characterized by hypertension and damage to multiple maternal organs, primarily the liver and kidneys. PE usually begins after 20 weeks’ gestation and, if left untreated, can lead to serious complications and lifelong disabilities—even death—in both the mother and the infant. As delivery is the only cure for the disease, treatment is primarily focused on the management of blood pressure and other clinical symptoms. The pathogenesis of PE is still not clear. Abnormal spiral artery remodeling, placental ischemia and a resulting increase in the circulating levels of vascular endothelial growth factor receptor-1 (VEGFR-1), also called soluble fms-like tyrosine kinase-1 (sFlt-1), are believed to be among the primary pathologies associated with PE. sFlt-1 is produced mainly in the placenta during pregnancy and acts as a decoy receptor, binding to free VEGF (VEGF-A) and placental growth factor (PlGF), resulting in the decreased bioavailability of each to target cells. Despite the pathogenic effects of increased sFlt-1 on the maternal vasculature, recent studies from our laboratory and others have strongly indicated that the increase in sFlt-1 in PE may fulfill critical protective functions in preeclamptic pregnancies. Thus, further studies on the roles of sFlt-1 in normal and preeclamptic pregnancies are warranted for the development of therapeutic strategies targeting VEGF signaling for the treatment of PE. Another impediment to the treatment of PE is the lack of suitable methods for delivery of cargo to placental cells, as PE is believed to be of placental origin and most available therapies for PE adversely impact both the mother and the fetus. The present review discusses the pathogenesis of PE, the complex role of sFlt-1 in maternal disease and fetal protection, and the recently developed placenta-targeted drug delivery system for the potential treatment of PE with candidate therapeutic agents.

## 1. Introduction

**Figure 1:**
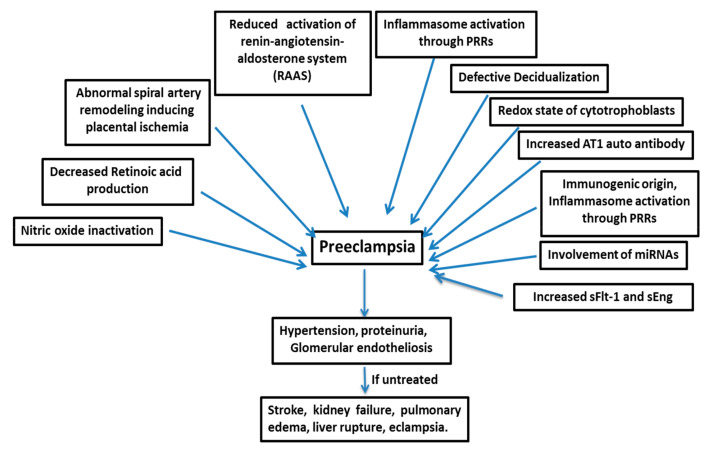
Preeclampsia is a multifactorial disease. sFlt-1=soluble fms-like tyrosine kinase-1, and sEng=soluble endoglin.

Hypertensive disorders are common pregnancy complications leading to serious outcomes for both mother and fetus. Preeclampsia (PE) is one of the most serious hypertensive disorders, affecting 5–7% of all pregnancies, and leads to about 70,000 maternal and 500,000 fetal deaths worldwide every year [1]. PE is also a leading cause of maternal death in the United States [2,3,4]. Moreover, preterm PE is associated with a greater risk of future cardiovascular and cerebrovascular diseases in both mothers and babies born to preeclamptic mothers [5,6]. PE is a multifactorial disease (Figure 1), and its pathogenesis is not attributable to a single factor, such as genetic, immunogenic, or environmental factors; rather, it is a disease manifested by a complex combination of different factors [7]. PE develops after 20 weeks of gestation with vascular dysfunction and, if untreated, leads to conditions like stroke, kidney failure, pulmonary edema, liver rupture, and eclampsia [8,9]. The only treatment adopted so far is the delivery of both fetus and placenta, which results in higher premature birth rates and restricted infant growth [10].

PE is broadly categorized into two types based on the gestational age at onset: placental PE (Type I, onset before 34 weeks’ gestation) and maternal PE (Type II, onset after 34 weeks’ gestation) [11]. Type I PE occurs due to the poor development of the placenta in early gestation, whereas Type II PE develops due to abnormal maternal responses towards the end of pregnancy. Recent large-scale microarray studies and the unsupervised clustering of PE patient samples revealed three distinct molecular subclasses of PE: (a) canonical, exhibiting established PE markers and molecular phenotypes; (b) immune response-related; and (c) the subclass representing poor maternal responses to pregnancy. These studies show PE to be a multifactorial disease with different molecular pathways marking each group [12,13]. A remarkable study by Than et al. (2018) using an integrated system biological approach showed the existence of distinct maternal and placental disease pathways in PE [14]. The maternal disease pathways are reflected in all phenotypes of PE, both upstream and downstream of placental dysfunction, whereas placental disease pathways are superimposed onto maternal disease pathways. These studies indicate the presence of distinct PE phenotypes and the likely need to target therapeutics against different groups of molecules in each case. Risk factors for PE include chronic hypertension, pregestational diabetes mellitus, obesity, antiphospholipid syndrome, and a history of PE [15].

**Figure 2:**
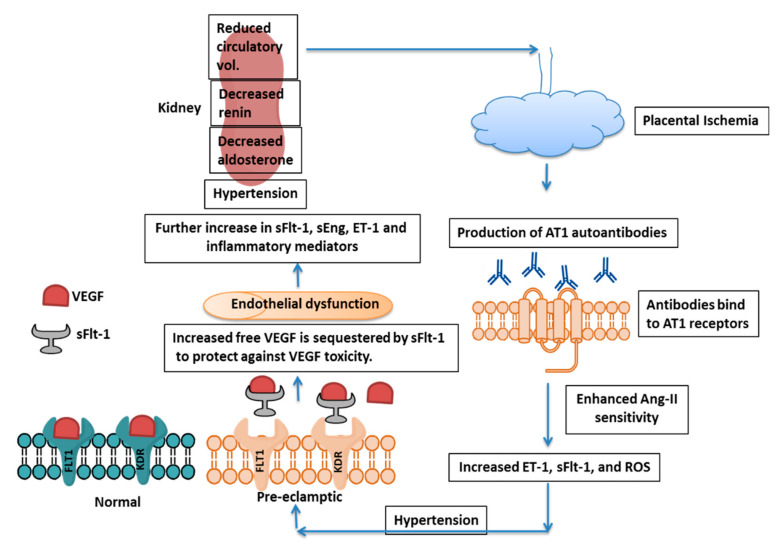
Mechanism of PE Pathogenesis. Placental ischemia induces AT1 autoantibody formation, which further increases Ang-II sensitivity, leading to enhanced ET-1, sFlt-1, and ROS production. sFlt-1 acts as a decoy receptor and blocks free VEGF to protect the fetus from toxicity by excess VEGF, although there is endothelial dysfunction and effect on kidney. AT1 = Angiotensin-II type 1, Ang-II = angiotensin-II, ET-1 = Endothelin-1, sFlt-1 = soluble fms-like tyrosine kinase-1, ROS = Reactive Oxygen Species, VEGF = Vascular Endothelial Growth Factor, KDR = Kinase insert Domain Receptor and sEng=soluble endoglin.

Although the etiology of PE is still not clear, conditions like defective decidualization, impaired cytotrophoblast invasion, endothelial dysfunction, and inappropriate immune responses to the allogenic fetus are thought to contribute to the disease. These processes are interconnected with a common downstream impairment of spiral artery remodeling and the excess release of soluble fms-like tyrosine kinase-1 (sFlt-1), the soluble form of the vascular endothelial growth factor (VEGF) receptor VEGF receptor 1 (VEGFR-1) into maternal circulation [16,17] (Figure 2). VEGF (VEGF-A) is mainly involved in endothelial cell proliferation, migration, and angiogenesis, and its function is mediated by two receptors: VEGFR-1 (also called Flt-1) and VEGFR-2 (also called kinase insert domain receptor (KDR)). Of these two, VEGFR-2 has higher tyrosine kinase activity, mediating the major mitogenic signals of VEGF, whereas VEGFR-1-mediated signaling plays a crucial role in angiogenesis in pathological conditions. VEGFR-1 is transactivated by the hypoxia-induced transcription factor Hif-2α, increasing angiogenesis, while Hif-1 also mediates enhanced sFlt-1 production in both in vivo and in vitro models [18]. Additionally, sFlt-1 and VEGF production have been shown to be regulated by adenosine in rat placental villous explants under hypoxic conditions [19].

The serum levels of VEGF in PE cases at 30 weeks of pregnancy are significantly higher compared to those in cases of gestational hypertension and healthy pregnancies, suggesting that VEGF acts as a marker for the diagnosis of this condition [20]. In turn, sFlt-1 sequesters the free form of VEGF and acts as an anti-angiogenic factor [21,22,23,24,25]. It has been suggested that immunogenic maladaptation occurs when fetal trophoblast cells invade the maternal decidua during implantation, resulting in reduced placental perfusion, failure of spiral artery remodeling, and abnormal placentation with resulting placental ischemia [26]. The ischemic condition further increases sFlt-1 and soluble endoglin (sEng) in maternal circulation, and thus there is vascular endothelial dysfunction, hypertension, and proteinuria.

Studies in our laboratory, along with recently published studies, have found that VEGF induces the expression of sFlt-1 in human vascular endothelial cells and placental trophoblast cells, while expression of full-length VEGFR-1 (Flt-1) is unaltered [25,27]. Although the precise mechanism is not fully understood, VEGF induction of sFlt-1 expression has been shown to be mediated by the VEGFR-2 protein kinase C-MEK signaling pathway [25]. This review article discusses the molecular mechanisms involved in the pathogenesis of PE, the delicate balance between VEGF and sFlt-1 in the disease process, and candidate therapeutic approaches.

## 2. Pathogenesis of Preeclampsia

Many factors are responsible for the pathogenesis of PE (Figure 1), such as the shallow invasion of cytotrophoblasts, impeded spiral artery remodeling, immune cells, genetics of the mother, involvement of microRNAs, etc.

### 2.1. sFlt-1: The Central Molecule in PE

sFlt-1 is a soluble splice variant of the full-length membrane receptor VEGFR-1, transcribed with premature polyadenylation of the VEGFR-1 transcript at intron 13 and encoding only the extracellular domain [28,29]. It lacks the seventh Ig-like domain, as well as the transmembrane and tyrosine kinase regions. Thus, it acts as a decoy receptor through binding and a reduction in free, circulating VEGF and placental growth factor (PlGF), thereby reducing their availability for angiogenesis [25,30,31]. Although its levels in maternal circulation increase dramatically in pregnancy, there is a further increase in PE, leading to the widely held notion that excess sFlt-1 is the primary cause of maternal vascular symptoms through its sequestration of VEGF [9,32,33]. Indeed, extracorporeal removal of sFlt-1 in maternal circulation alleviates the maternal symptoms [34], although these studies have not directly evaluated the potential negative consequences for the fetus in removal of sFlt-1, which has been shown to protect the fetus from the toxic effects of excess VEGF in mouse models. Thus, understanding the causes of sFlt-1 upregulation is central to understanding the disease and may lead to effective treatments.

Trophoblast cells are the primary producers of the sFlt-1 that enters maternal circulation during pregnancy [35]. The level of sFlt-1 in serum is found to be 20–50 times higher in healthy pregnancies as compared to the non-pregnant state, with peak concentrations at term, suggesting its importance in normal pregnancy [36]. It acts as a regulator of placental invasion in healthy pregnancies, maintaining the placental position at an appropriate depth in the uterine wall [37], although one study showed trophoblast-derived Flt-1/sFlt-1 is dispensable in the establishment of pregnancy in mouse placenta [38].

The mechanism of sFlt-1 splicing in placental hypoxia is explained in a recent study by Palmer et al. (2016) [39]. It was observed that the oxygen-sensing protein Jumonji domain-containing protein 6 (JMJD6) influences the splicing of Flt-1 in endothelial cells [40]. JMJD6 is an oxygen-sensing protein belonging to the 2-oxoglutarate-dependent oxygenase superfamily [41]. This protein depends on the availability of oxygen for its catalytic activity [42,43]. The JMJD6 protein hydroxylates a component of the splicing machinery, U2 small nuclear ribonucleoprotein auxiliary factor 65 kDa subunit (U2AF65), under normoxic conditions, and full-length membrane-bound Flt-1 transcript is produced [40]. However, in hypoxic conditions, JMJD6 activity is reduced, and thus the level of the unhydroxylated form of U2AF65 is increased, with enhanced production of shorter, alternatively spliced sFlt-1 transcripts from the Flt-1 primary transcript.

Palmer et al. (2017) observed that JMJD6 is associated with splicing of both the sFlt-1 i13 and sFlt-1 e15a isoforms of sFlt-1, and the loss of JMJD6 in hypoxic conditions leads to greater production of the sFlt-1 e15a variant, a marker of PE [44]. The sFlt-1 e15a isoform (present in humans and higher-order primates) seems to be the predominant form, found in abundant amounts in placenta and responsible for endothelial and end-organ dysfunction in PE [44,45]. However, a recent in vivo study showed that overexpression of the full-length hsFlt-1 e15a isoform promotes increase in litter sizes in mice and regulates embryonic development [46].

Excess sFlt-1 is thought to cause maternal symptoms of PE. In preeclamptic placentas at 11 weeks of pregnancy, sFlt-1 production is increased significantly, with increased expression beginning 5 weeks before the onset of clinical symptoms [47]. Analysis of supernatants of preeclamptic placental villous explants revealed a 4-fold increase in sFlt-1 levels over those from normal pregnancies [48]. A study by Powers et al. (2005) revealed that maternal serum sFlt-1 concentrations are not increased in early pregnancy in women who develop PE [49]. This molecule might play a role in oxidative stress and apoptosis in trophoblasts, as there was upregulation of apoptosis markers in sFlt-1-treated mice, along with mitochondrial swelling, suggestive of mitochondrial apoptotic pathway activation [50]. sFlt-1 has an inhibitory effect on placental vascularization through VEGF signaling reduction, leading to apoptosis of fetal vessels and impaired placental differentiation and nutrient exchange, which ultimately results in fetal growth restriction, mimicking the PE condition.

VEGF-A and PlGF play crucial roles in angiogenesis in the fetus during pregnancy. Placental hypoxia stimulates the production of these angiogenic factors as well as their endogenous inhibitor, sFlt1, and an imbalance in the production of these factors may lead to PE [51]. Although VEGF-A expression is essential for vasculogenesis/angiogenesis in the embryo, even a 2–3-fold increase in its expression disrupts embryonic development in mice [52]. Using endometrium-specific VEGF-A overexpression in a mouse model, a recent study from our group revealed that increased production of VEGF-A in the endometrium elicited an increase in placental production of sFlt-1 and resulted in PE-like symptoms in the mother. VEGF-A overexpression also caused placental vascular defects and fetal loss, indicating that limiting VEGF levels is critical to placental development and fetal survival. Moreover, knockdown of sFlt-1 in otherwise wild-type mice caused similar placental vascular defects with fetal loss, and knockdown of placental sFlt-1 in endometrial VEGF-overexpressing mice exacerbated the vascular defects caused by VEGF overexpression alone [27]. This study indicates that VEGF overproduction might play a role in PE, but more critically, it shows that sFlt-1 plays an essential protective role in normal pregnancy and that increased sFlt-1 in PE is a regulatory response to protect the placenta and fetus from VEGF toxicity. An in vitro study using murine trophoblast stem cells also revealed increase in sFlt-1 mRNA levels in response to VEGF. Another study by Murakami et al. (2005) showed VEGF playing a major role in the pathophysiology of PE, as exogenous murine VEGF164 induced PE-like symptoms in pregnant mice, causing hypercoagulation in the placental circulation and elevation of blood pressure [53]. In a similar fashion, a study by Parchem et al. (2018) revealed that loss of PlGF is a protective measure against the development of PE, which contradicts the accepted notion that increased sFlt-1 causes PE by inhibiting PlGF and VEGF function [54]. This study in PlGF knockout mice found that the mice did not develop signs of PE, despite elevation of circulating sFlt1 levels. However, the PlGF knockout mice did exhibit morphological changes in the placenta, with abnormal accumulation of junctional zone glycogen [54].

ELABELA (also known as ELA or Apela or Toddler) is a peptide hormone of 32 amino acids that acts primarily by binding the apelin receptor (APLNR or APJ). It is found to be expressed in endothelial cells, kidney, and prostate, besides its critical role in embryonic development [55,56]. ELA is important for early placentation and embryonic cardiovascular development, whereas apelin acts in mid or late stage pregnancy to modulate fetal angiogenesis and energy homeostasis. Apelin is produced from preproapelin (77 amino acids long in human) and has many isoforms. The apelin-APLNR system in adults promote angiogenesis, vasodilatory effects, cardiac contractility, glucose uptake, etc. [57,58,59]. Both the ELA and apelin hormone show their function in the fetoplacental unit through their common APLNR receptor. Pathway studies on HTR8/SVneo cell line (human extravillous trophoblast cells used for in vitro studies) and human placental explants (first trimester) revealed the ability of ELA to enhance invasion of EVTs (extravillous trophoblasts) and thus support healthy pregnancy [60,61]. Moreover, it has been found that the level of ELA in circulation in pregnant women is correlated with BMI (body mass index), i.e., ELA levels are lower in women with healthy BMIs in the first trimester but who develop PE in later stages of pregnancy. One study in mice showed the development of PE in response to a deficiency of ELA, but a human study showed no differences in ELA levels between control and PE patients. However, the level of apelin is increased in PE in the later stages of pregnancy, as reviewed by Eberle et al. (2019) [62]. A study by Zhou et al. (2019) showed ELA levels to be significantly reduced in late-onset PE, suggesting a role in the pathogenesis of this type of PE [63]. Further study is needed to understand the involvement of ELA in the pathogenesis of PE in humans.

### 2.2. Factors Regulating sFlt-1 Expression

Many factors have been found to regulate sFlt-1 expression directly or indirectly through various signaling pathways. Expression levels of sFlt-1 and VEGF-A were measured in HTR-8/SV neo and JEG3 cells transfected to express exogenous VEGF165 [64]. Elevated VEGF-A expression was found to upregulate the production of sFlt-1 and reduce the migration and invasion capability of trophoblast cells. Interestingly, the sFlt-1 level decreased significantly, and there was rescue of the migration and invasion defect of trophoblast cells upon treatment with VEGF-A receptor inhibitors. These findings indicate that sFlt-1 upregulation in response to VEGF-A might be mediated by the VEGF/Flt-1 and/or VEGF/KDR signaling pathways.

Retinoic acid (RA) seems to regulate sFlt-1 expression negatively in decidual stromal cells, as preeclamptic decidual cells showed lowered levels of RA. RA suppresses sFlt-1 expression without affecting decidualization, and decreased RA levels may promote PE due to resulting sFlt-1 accumulation at the maternal-fetal interface [65]. Moreover, nitric oxide (NO, a vasodilator derived from nitrite) prevents hypertension during pregnancy and causes a reduction in the circulating plasma levels of sFlt-1 and VEGF, and NO inactivation leads to endothelial dysfunction, culminating in PE [66]. Glomerular endotheliosis induced in pregnant mice by adenovirus-mediated overexpression of sFlt-1 developed albuminuria and hypertension [67]. Glomerular endothelial NO generation is impeded in sFlt-1-induced PE in pregnant mice because cationic amino acid transporter-1 (CAT-1) is inhibited by pregnancy and PE through phosphorylation. L-arginine was found to prevent blood pressure increases and albuminuria and thus reduce the complications arising from PE [67]. Mice lacking the G protein-coupled receptor kinase interactor 1 (GIT1) gene (GIT-/-) were found to have reduced NO production. GIT1, a GTPase-activating protein, plays a crucial role in regulating the biological activity of endothelial nitric oxide synthase (eNOS) [68]. The absence of GIT1 seems to aggravate PE-like phenotypes in mice by impeding NO production through suppression of eNOS activity [69].

An in vitro study with BeWo placental trophoblast cells showed transcript levels of sFlt-1 were the same in hypoxic and normoxic conditions, whereas the release of sFlt-1 into the media was higher in hypoxia. sFlt-1 was found to bind to the heparan strands present in the extracellular matrix (ECM) through its heparin-binding site. These strands are cleaved by extracellular heparanase, which was found to be expressed at higher levels in hypoxic conditions. These findings indicate heparanase might play a role in the release of sFlt-1 in disease conditions like PE [70]. Human endometrial stromal cells produce detectable amounts of sFlt-1, and this expression is turned off at both the protein and RNA levels during the decidualization of stromal cells. sFlt-1 expression seems to be negatively correlated with prolactin (a decidualization marker) and VEGF expression in endometrial stromal cells [71]. It has been found that the protein growth arrest and DNA-damage-inducible 45alpha (Gadd 45α), is highly expressed in preeclamptic placentas, and its expression is induced by oxidative stress. This protein enhances the levels of sFlt-1 and sEng by increasing the activities of matrix metalloproteinases 2 and 9 (MMP-2/9), mediated by the P38 MAPK (mitogen activated protein kinase) signaling pathway [72].

### 2.3. Impeded Spiral Artery Remodeling

Most studies of PE have focused on the placenta as the main agent in the development of the disease; however, recent findings show that abnormal interactions between the placenta and uterus leads to the serious condition. There is inadequate spiral artery remodeling and shallow implantation in the uterine wall. The initial event in PE is found to be reduced utero-placental perfusion due to abnormal trophoblast invasion of spiral arterioles. The resulting placental ischemia caused by impaired spiral artery remodeling results in the increased production of sFlt-1, Ang II type 1 autoantibodies, TNF-α, etc., which in turn cause maternal vascular endothelial dysfunction and PE [73,74]. One study revealed that TNF-α stimulates sFlt-1 production in response to placental ischemia in rats [75]. Abnormal spiral artery remodeling induces placental ischemia and affects the biological activity of mitochondria, and thus reactive oxygen species (ROS) are produced. ROS ultimately stabilize Hif-1α, which might induce sFlt-1 and sEng production. Thus, mitochondrial dysfunction (leading to oxidative stress) plays a vital role in increased sFlt-1 production [76]. One study showed mitochondrial function can be improved by treatment with AP39 (a mitochondrion-targeted hydrogen sulfide donor), which ultimately reduced sFlt-1 levels by decreasing ROS levels and Hif-1α production [77]. Thus, mitochondria-targeted antioxidants can be used as therapeutics to alleviate PE symptoms. Defects in maternal stromal cell decidualization may also lead to abnormal overexpression of sFlt-1 and disease [16]. Endometrial stromal cells (ESCs) undergo decidualization with change of shape and function, secreting factors like IGFBP1 and prolactin, which promote implantation and placental development [78,79,80]. Decidual stromal cells (DSCs) isolated from PE placentas are unable to respond adequately to in vitro decidualization induction as compared to those of normotensive DSCs and ESCs from non-pregnant women. Additionally, the stimulated redecidualized cells from PE placentas downregulated sFlt-1 production less efficiently than the other two cell types [16].

Impairment of spiral artery remodeling causes a decrease in placental perfusion, resulting in placental ischemia and sFlt-1 release. sFlt-1 binds to free PlGF and VEGF and thus sequesters these factors resulting in endothelial dysfunction. The endothelin system also plays crucial role in response to placental ischemia and promotes hypertension and PE condition (reviewed by LaMarca et al. (2008) [81]. Physiological dysfunction in the endothelium triggers the production of endothelin-1 (ET-1), which further induces hypertension and proteinuria and suppresses renin release. The rise in blood pressure also suppresses renin activity, which is accompanied by aldosterone suppression. This reduced activation of the renin-angiotensin-aldosterone system (RAAS), along with high blood pressure, causes a reduction in circulatory volume, which further decreases placental perfusion [82]. It is reported that protease activated receptor-I (PAR-I), a member of the G protein-coupled protease activated receptor family, acts as a thrombin receptor, and thrombin increases sFlt-1 expression by trophoblasts through the PAR-I receptor [83]. It seems that inhibition of trophoblast PAR-I overexpression causes suppression of sFlt-1-induced anti-angiogenesis and abnormal vascular remodeling [84]. Use of PAR-I inhibitors may therefore be a valid therapeutic approach to control preeclampsia.

MMPs include enzymes like collagenases, gelatinases, stromelysins, etc., which play crucial roles in tissue remodeling by degrading various proteins in the ECM [85,86,87]. MMPs are found to regulate spiral artery remodeling during pregnancy, and reduced levels of MMP-2 (gelatinase A) and MMP-9 (gelatinase B) might be responsible for reduced vasodilatation, enhanced vasoconstriction, and the development of PE [88]. These two enzymes are adequately produced by EVTs and degrade the ECM and thus promote the EVT invasion and spiral artery remodeling [89,90,91]. The immunoglobulin superfamily member EMPRIN has been observed to play role in the elevation of vascular MMPs induced by the female sex hormones estrogen and progesterone [92]. The activities of MMPs can be modulated by tissue inhibitors of metalloproteinases (TIMPs) and bring changes in the MMP/TIMP ratio. MMP gene expression is also regulated by various extracellular cytokines and growth factors like EGF, TNF-α, IFN-γ, TGF-β, etc. [93]. IFN-γ, along with TNF-α, are found to inhibit EVT invasion through a reduction in pro-MMP-2 secretion and enhanced uPA secretion [94]. Uterine natural killer (NK) cells are the major source of IFN-γ, and this cytokine inhibits EVT invasion through increased EVT apoptosis and decreased MMP levels, leading to the PE condition [95].

### 2.4. Redox State of Cytotrophoblasts

The redox state in placental cytotrophoblasts plays a crucial role in regulating blood pressure during pregnancy. K_Ca_2.3 and K_Ca_3.1 are the K^+^ channels involved in the endothelial control of vascular contractility [96]. Altered redox states in cytotrophoblasts modulate the expression levels of endothelial K_Ca_2.3 and K_Ca_3.1, and thus affects vascular contractility. These Ca^2+^-activated K^+^ channels induce NO release by promoting Ca^2+^ influx through Ca^2+^ entry channels. Thus, the upregulation of K^+^ channels facilitates endothelium-dependent relaxation, whereas their downregulation results in vasoconstriction and blood pressure elevation [97,98]. In PE there is downregulation of K_Ca_2.3 and K_Ca_3.1 due to superoxide generation resulting from superoxide dismutase (SOD) downregulation and NADPH oxidase (NOX) upregulation, ultimately promoting hypertension [96]. Levels of the trace element strontium are higher in PE patients, along with uric acid, and there is alteration in the redox state in cells of preeclamptic women. These findings indicate the involvement of strontium in the pathogenesis of PE [99].

### 2.5. Angiotensin-II Type 1 Receptor Autoantibody

Angiotensin-II type 1 receptor (AT1) is found to play a crucial role in the pathophysiology of PE. Angiotensin-II binds to this receptor and induces vasoconstrictive activity. Women with PE have increased concentrations of AT1 autoantibodies, sFlt-1, and sEng, and increased sensitivity to angiotensin-II [100]. sFlt-1 and angiotensin-II levels are significantly increased in preeclamptic rat models, with angiotensin-II promoting sFlt-1 production in response to placental ischemia. Administration of the AT1 receptor antagonist losartan reduced plasma sFlt-1 levels and hypertension in a reduced uterine perfusion pressure (RUPP) model in rats [101]. Angiotensin 1-7 (Ang 1-7) is found to reduce the symptoms of PE, which might occur through increases in PPAR-Υ expression and the lowering of asymmetric dimethylarginine levels [102].

### 2.6. Inflammatory Cytokines in PE Pathogenesis

Immune cells play a vital role in the pathogenesis of PE. Studies in our laboratory revealed a role for macrophages in regulating pregnancy and the development of PE, as the M2 phenotype (anti-inflammatory) was found to predominate in normal pregnancy and the M1 phenotype (inflammatory) was found to predominate in PE [103]. Macrophage recruitment in the decidua and polarization is largely induced by VEGF. The dynamic function of macrophages in normal pregnancy and different complications, including PE, have been reviewed thoroughly by Jena et al. (2019) [104].

NK cells constitute 70% of the leukocytes present in the endometrium during early pregnancy and play vital roles in the processes of implantation and placental development [105]. The interaction of killer cell Ig-like receptors (KIRs) on NK cells with HLA-C ligands present on trophoblasts induces NK cells to secrete angiogenic cytokines required for trophoblast invasion and vascular remodeling. The abnormal activation of both peripheral NK (pNK) and decidual NK (dNK) cells leads to the development of PE. dNK cells play vital roles in trophoblast invasion and spiral artery remodeling [106]. These cells promote trophoblast invasion by producing the chemokines CXCL8 and CXCL10, which interact with receptors such as CXCR1 and CXCR3 [107,108]. dNK cells produce IFN-γ, which further enhances the production of CXCL9, CXCL10, CCL8, and CCL5 by dNK cells. These cytokines promote trophoblast invasion and spiral artery remodeling in successful pregnancy. Moreover, dNK cells produce VEGF, angiopoetin-2, and PlGF, which promote successful placentation and fetal angiogenesis [95,109,110]. In PE, CXCL10, CXCL8, CCL2, and CCL5 levels increase relative to levels in normal pregnancy, creating a proinflammatory environment [111,112,113]. dNK cells also facilitate EVT motility by secreting hepatocyte growth factor [114]. The impairment of the interactions between dNK cells and trophoblasts results in poor placentation and an enhanced risk of PE [115].

CD4^+^ T cell number is found to increase in PE, along with the levels of the inflammatory cytokines IL-17 and TNF-α, whereas regulatory T cell number decreases, and the levels of anti-inflammatory cytokines are reduced. A comparative study of the effects of placental CD4+ T-cells from a PE patient and those from normal pregnancies introduced into nude athymic rats revealed an association of these T cells with the pathogenesis of PE [116]. The level of IL-17 and TNF-α was increased in the recipients of PE-associated CD4+ T cells. The enhanced T-cell population also induces B cells to produce agonistic AT1 autoantibodies (AT1-AA) that activate the AT1 receptor, which ultimately regulates vasoactive factors [117]. A study of the associations among placental lesions, cytokines, and angiogenic factors found increased syncytial knots and perivillous fibrin deposits in early-onset PE placentas relative to those from normotensive and late-onset PE. The TNF-α/IL-10 and sFlt-1/PlGF ratios were also greater in placental homogenates of early-onset PE than those from late-onset PE and normotensive women [118].

The level of CRP, an acute innate immune mediator, is increased in the circulation before the onset of symptoms of PE. CRP production is mainly stimulated by the cytokines IL-6, IL-8, and TNF-α, but the increased levels in PE might be caused by neurokinin B-induced activation of the neurokinin 3 receptor [119]. The placenta-specific enzyme phosphocholine transferase makes a posttranslational modification of neurokinin B, which ultimately induces the increase in expression of CRP in preeclamptic women. Furthermore, CRP is a potential biomarker for early diagnosis of PE due to its early rise in the maternal circulation. A study using an ultrasensitive assay revealed increased levels of inflammatory markers, such as CRP and IL-6, in preeclamptic pregnancies as compared to age-matched normal pregnancies. This indicates the involvement of CRP and IL-6 in the pathogenesis of PE and warrants further study [120].

Complement activation is associated with renal dysfunction, and there is an accumulation of markers of complement activation, such as C4d and C1q in PE; hence, the inhibition of complement activation may be helpful in controlling the renal manifestations of PE [121]. Increased urinary C5b-9 levels in PE women were found to be potential biomarkers that distinguish PE from other hypertensive disorders [122,123]. A study on the HTR-8/SVneo cell line showed C5a induced higher levels of sFlt-1 transcription [124].

Pattern recognition receptors (PRRs) present in the cell recognize pathogens and damaged tissues to initiate inflammatory responses. The activation of the nod-like receptor protein (NLRP3) inflammasome through PRR is induced by danger signals, like the crystalline form of cholesterol and uric acid, and leads to the activation of the proinflammatory cytokine IL-1β [125,126,127,128]. PE has been found to be associated with increased cholesterol and uric acid levels, and the mechanism of inflammation in PE is primarily through NLRP3 inflammasome activation in trophoblasts [129]. NLRP3 inflammasome pathway components, such as NLRP3, caspase-1, and IL-1β, were shown to be co-expressed with priming factors, like complement factor C5a and terminal complement complex (TCC), in the syncytiotrophoblast. This mechanism may contribute to placental inflammation and the pathogenesis of PE. Additional immunological aspects of PE development include changing paternity, shorter sexual cohabitation periods, and nulliparity [130,131].

Hyperglycemia has effects on the functionality of trophoblasts and early placentation, as excess glucose induces the inflammation of trophoblasts. The inflammatory condition limits trophoblast migration through the activation of TLR-4 by the damage-associated molecular pattern (DAMP) protein high-mobility group box-1 (HMGB-1) protein. Hyperoxia (rather than hypoxia or normoxia) is the main driver of trophoblast dysfunction in response to excess glucose [132].

### 2.7. Signaling Pathways Involved

Many signaling pathways have been shown to play vital roles in the pathogenesis of PE. Placental oxidative stress induces the secretion of anti-angiogenic factors, like sFlt-1 and sEng, while the nuclear factor erythroid 2-like 2 (Nrf2) pathway is one of the most important pathways for protecting the cell from oxidative stress. Nrf2 acts as a crucial inducer of genes encoding detoxification enzymes and antioxidative proteins, and it restores the balance between pro- and anti-angiogenic factors, mainly through the downstream target protein heme oxygenase-1 (HO-1). HO-1 primarily metabolizes heme to biliverdin, iron, and carbon monoxide (CO), and CO promotes an increase in VEGF synthesis, ultimately causing vasodilatation and attenuating hypertension. HO-1 was also found to inhibit the production of sFlt-1 in an in vitro study. The Nrf-2/HO-1 pathway plays an important protective role in PE and can be targeted for the treatment of this disease [133,134].

Activation of the mammalian target of rapamycin (mTOR) signaling pathway, which regulates proliferation, autophagy, and cell death, is found to improve the pregnancy outcomes of rats induced by N-carbamoyl glutamate [135]. Conversely, the suppression of the mTOR pathway induces PE through a decrease in the invasiveness of trophoblast cells [136]. Recent studies have also revealed that the silencing of G protein γ 7 (GNG7), a regulatory G protein, suppresses apoptosis in preeclamptic rats via activation of the mTOR signaling pathway, thus enhancing the proliferation and differentiation of cytotrophoblasts [137].

Low-dose aspirin (LDA) is found to reduce the hypoxia-induced release of sFlt-1 in trophoblast and endothelial cells, and this activity is mediated through the c-Jun NH2-terminal kinase/activator protein-1 (JNK/AP-1) pathway. The transcription factor AP-1 regulates sFlt-1 production by binding to the Flt-1 promoter. LDA mitigates the hypoxia-induced apoptosis of trophoblasts, and thus trophoblast migration and invasion capabilities are restored [138].

### 2.8. The Role of miRNAs and lncRNAs in Pathogenesis

miRNAs (small 18–24 nt noncoding RNAs) regulate gene expression post-transcriptionally by binding to the 3′-UTRs (3′-untranslated regions) of mRNAs [139]. Aberrant expression of miRNAs has been reported in many disease conditions, including PE, and some miRNAs are discussed here. miR-19a overexpression protects endothelial cells from apoptosis (induced by lipopolysaccharides) and increases cell invasion and metastasis [140,141]. These observations indicate the involvement of miR-19a in the development of PE. The miRNA miR-26a-5P is found to be expressed and secreted through urine in preeclamptic patients suffering from proteinuria and podocyturia. Deile et al. (2018) demonstrated in a preeclamptic model in Zebrafish that miR-26a-5P is upregulated in PE, targeting podocyte VEGF-A [142]. The glomerular changes observed are edema, proteinuria, glomerular endotheliosis, and podocyte effacement, resembling findings in human PE. This miR has the potential to act as biomarker for early diagnosis of PE. miR-195-5P levels were found to increase in correlation with levels of sFlt-1 in preeclamptic pregnant women [143]. Additionally, some protein targets of miRNAs have been found to increase in PE. Pregnancy-specific glycoproteins (PSGs), which are of trophoblastic origin are found in maternal blood during pregnancy [144]. PE placentas have higher levels of PSG10P (a target of miR-19a-3P) and lower levels of miR-19a-3P as compared to normal placentas [145]. Additionally, there are increased levels of IL-1RAP proteins (targets of miR-19a-3P) in PE placentas. Therefore, there might be a regulatory network existing in the PSG10/miR-19a-3P/IL-1RAP pathway which may contribute to the pathogenesis of PE [145].

Lin28 is an RNA-binding protein which plays vital roles in cellular differentiation, invasion, and growth of the embryo [146]. Of the two Lin28 paralogs (Lin28A and Lin28B), Lin28B is expressed at 1300-fold greater levels than Lin28A in human term placentas, but its expression is significantly lower in PE. miR Let-7 regulates Lin28 expression and lowered Lin28B expression results in reduced trophoblast invasion and induces inflammation and PE [147].

The placenta-specific miRNAs, which are located in the chromosome 19 miRNA cluster (C19MC) are found to be associated with the pathogenesis of PE. One study revealed that the 10 placenta-specific miRNAs (miR-518b, -1323, -516b, -516a-5P, -525-5P, -515-5P, -520h, -520a-5P, -519d and -526b) present in the C19MC are found at greater levels in the plasma of PE patients than that of normal pregnant women [148]. This observation suggests the involvement of C19MC in PE pathogenesis.

lncRNAs (long non-coding RNAs of >200 nt), which regulate gene expression by acting as coregulators or through complementary binding, may also be involved in PE [149]. The expression of the lncRNA PSG10P (a noncoding pseudogene) was significantly upregulated in PE placentas, suggesting its possible importance in the condition [150]. Pregnancy-specific glycoproteins (PSGs), which are of trophoblastic origin, are found in maternal blood during pregnancy [144].

### 2.9. The Role of Endothelin-1 (ET-1) in the Pathophysiology of PE

ET-1 is a potent vasoconstrictor produced and secreted mainly by endothelial cells. It acts primarily in an autocrine fashion, but under certain physiological conditions, it can affect distant organs through entry into the systemic circulation [151,152]. ET-1 acts by binding to two cell surface G-protein coupled receptors: ET-1 type-A (ET_A_) and type B (ET_B_). ET-1 may be involved in the pathophysiology of PE, as its levels are elevated in PE. Other studies have also shown increased levels of pre-pro-ET-1 mRNA at the tissue level [153]. Additionally, experimental PE models have shown good responses to ET_A_ antagonism for amelioration of the disease, warranting further studies. ET_A_ antagonists could decrease hypertension and be used as therapeutic agents in PE [154].

sFlt-1 and ET-1 act in complementary fashion in the pathogenesis of PE [155]. The diminished perfusion of the placenta resulting from improper spiral artery remodeling induces the production of sFlt-1 as a consequence of placental ischemia. sFlt-1 acts as a decoy receptor for free VEGF, thus affecting angiogenesis and inducing endothelial dysfunction. ET-1 production is initiated by endothelial dysfunction and further promotes hypertension and proteinuria while suppressing renin release. There is simultaneous suppression of aldosterone levels, and this reduced renin-angiotensin-aldosterone level culminates in a reduced circulating blood volume, further aggravating PE by reducing placental perfusion. In addition, ET-1 induces sFlt-1 production by the placenta and further complicates PE [82,155].

### 2.10. Miscellaneous Factors

Many other factors, such as the genetics of the mother (reviewed by Tomimatsu et al. 2019) [156], hormonal imbalance, neurotransmitter levels, and misfolded protein accumulation also play a role in the pathogenesis of PE.

African American mothers have a greater risk of developing PE than white mothers [157]. Much of this risk may be attributed to the fetal APOL1 genotype. APOL1 (encoding apolipoprotein 1) has two genetic variants—G1 and G2—found exclusively in people of African origin. Mothers carrying any combination of these two alleles are at greater risk of developing PE [158]. In-depth study is needed to determine whether fetal APOL1 genetic analysis can predict PE. In addition, trisomy of chromosome 13 (containing the Flt-1 gene) is associated with increased sFlt-1 production and a higher risk of PE [159]. A cohort study revealed daughters born from preeclamptic pregnancies have higher risks of developing PE, and women who become pregnant by men born from preeclamptic pregnancies also have an increased risk [160,161]. A genome-wide study showed that a single-nucleotide polymorphism near the Flt-1 locus of the fetal genome is associated with the development of PE in the mother [162,163].

The placental hormone human chorionic gonadotropin (hCG) is produced by trophoblast cells and regulates embryo implantation, immune cell activity, progesterone production, and uterine development [164]. Higher levels of hCG are harmful to pregnancy in early stages, enhancing the risk of PE [165]. A 1.5- to 2.7-fold greater risk of PE was observed with high hCG concentrations and increased sFlt-1/PlGF ratios. It has also been observed that the neuropeptide Y (NPY, a sympathetic neurotransmitter), produced at higher levels in response to stress, is increased in PE. NPY accumulates in platelet cells, which are also increased in number, and can act as a vasoconstrictor [166]. NPY is a potential target for anti-hypertensive therapy in PE.

The endoplasmic reticulum (ER) is the site of protein folding and assembly, and when the protein load exceeds the capacity of the ER folding machinery, misfolded or unfolded proteins accumulated in the ER. The cell has a protection mechanism to overcome misfolded protein accumulation—the unfolded protein response (UPR) [167,168]. If the UPR mechanism fails to overcome the stress of misfolded protein formation, the apoptotic pathway is triggered to eliminate the cell. The role of protein misfolding in the pathogenesis of PE has been thoroughly reviewed by Gerasimova et al. (2019) recently [169]. It has been found that protein misfolding and aggregation plays a role in the pathogenesis of PE. Many proteins, like amyloid beta peptide, transthyretin, alpha-1 antitrypsin, albumin, and ceruloplasmin are misfolded, and the amyloid-like aggregates are found in the placenta, as well as in body fluids. These aggregates appear to induce ER stress, impaired trophoblast invasion, and placental ischemia, which culminates in PE condition [169].

## 3. Animal Models Used to Study PE

Various mouse and rat models have been used to study the pathophysiology of PE, some of which are discussed here. The RUPP model is mainly used in rats [170,171], baboons [172], and mice [173]. A novel RUPP model of PE in mice was developed by Fushima et al. (2016), which showed hypertension, glomerular endotheliosis, proteinuria, and fetal growth restriction associated with placental ischemia [174]. This models human PE and will enable genetic approaches to unravel the pathogenesis of PE and develop possible therapies. Huang et al. (2014) have generated a rat eclampsia-like model using pentylene tetrazol (PTZ) in an established rat preeclampsia model [175]. This eclamptic model suggests an inflammatory mechanism plays an important role in PE. In another model in mice, a doxycycline-inducible transgenic human sFlt-1 was used to induce stable and reproducible human sFlt-1 expression in the mouse. Fetal growth restriction is one of the important manifestations of PE, and it was observed that the induction of hsFlt-1 enhanced serum sFlt-1 and sFlt-1 mRNA levels in both the placenta and fetuses (heterozygous and homozygous fetuses), causing fetal growth restriction [30]. A method of local gene delivery using adenoviruses targeting mouse endometrium was developed in conjunction with non-invasive imaging (live bioluminescent imaging) of expressed transgenes [176]. This method is highly useful for studies involving gene-specific functions in the endometrium. Pregnancy stage-specific induction of gene expression in the placenta and live monitoring of gene expression during pregnancy is helpful for studies of placental development, pregnancy, and pregnancy complications like PE [177]. An ApoE knockout mouse model of PE was established to explore the pathogenesis of PE resulting from abnormal lipid metabolism and vascular endothelial injury [178]. An experimental mouse model using lentiviral vector-mediated placenta-specific expression of human sFlt-1 revealed preeclamptic symptoms along with IUGR. The drug pravastatin induced PlGF production and ameliorated the PE symptoms in the mouse model [179].

## 4. Therapeutic Approaches

Various therapeutic reagents have been predicted to be effective in managing the preeclamptic condition, although delivery of the placenta is the only solution to relieve maternal hypertension and proteinuria.

In a human case of very early-onset PE (15 weeks of gestation), sFlt-1 apheresis was performed 13 times in of the interval from 19 to 23 weeks of gestation; however, at 23 weeks and 3 days-cesarean section had to be performed due to maternal respiratory failure and fetal distress. This study indicates sFlt-1 functions to protect the placenta and fetus in PE and that removal may have negative consequences [180].

A study in a rat model of PE induced by NG-nitro-Larginine-methyl ester (L-NAME) to evaluate the therapeutic effect of quercetin (a bioflavonoid having antioxidant and reno-protective properties) in combination with aspirin showed amelioration of symptoms through a reduction in sFlt-1 and VEGF levels in the uterus [181]. Moreover, the prenatal treatment of preeclamptic animal models with pravastatin (a drug of choice to lower bad cholesterol) improved blood pressure, vascular activity, and pup growth and led to an increase in VEGF and PlGF levels and a decrease in sFlt-1 levels [179,182,183,184,185].

The drug sulfasalazine (an anti-inflammatory and antioxidant), used to treat autoimmune diseases and found to be safe during pregnancy [186] was shown to reduce sFlt-1 and sEng levels and increase PlGF secretion from human placenta [187]. Additionally, sulfasalazine, which mitigates endothelial dysfunction, a major pathologic condition in PE could be used as a therapeutic agent for PE but needs further investigation. A study to determine the therapeutic efficacy of relaxin (serelaxin, i.e., recombinant human relaxin-2) for the treatment of PE revealed this hormone reduces blood pressure and the levels of circulating TNF-α, sFlt-1, and preproendothelin while simultaneously increasing NO bioavailability in RUPP rats [188]. In PE, there is a decreased level of vasodilators, like NO, and increased levels of vasoconstrictors, like ET-1 [73,189,190,191,192]. Relaxin is a protein hormone of 6 kDa MW produced by ovaries (corpora lutea), cells of non-pregnant endometrium, decidual cells of pregnant endometrium, and blood vessels, cytotrophoblasts, and syncytiotrophoblasts [193]. In addition to its role in the relaxation of skeletal soft tissue and the cardiovascular and renal systems, relaxin plays an important role in maintaining blood pressure in normal pregnancy and is found to reduce blood pressure in a rodent model of hypertension [194].

Fasudil is a first-generation Rho/Rho-associated protein kinase (ROCK) inhibitor frequently used for the treatment of hypertension and other cardiovascular diseases [195]. A study by Gu et al. (2017) revealed that fasudil can attenuate hypertension induced by sFlt-1 in preeclamptic mice through inhibition of the RhoA/ROCK pathway [196]. Rho GTPases play crucial roles coupling the cellular redox state to endothelial cell function [197]. RhoA (Ras homolog gene family, member A) proteins are expressed at higher levels in PE, suggesting a role in PE pathogenesis [198]. Antioxidants like vit-C and vit-E inhibit the p38 signaling pathway and thus block sFlt-1 secretion in hypoxia-reoxygenation-induced endothelial cell monolayers [199].

Studies have also revealed that exogenous alpha-1 anti-trypsin can alleviate hypoxia/reoxygenation injury by reducing oxidative stress through inactivation of Rac1/p38 signaling [200]. Eddy et al. (2018) reviewed the use of VEGF and PlGF as therapeutics to curb PE and suggested the modified stabilized members of the VEGF family could be used as therapeutic agents for treatment [201], but recent studies reveal VEGF and PlGF could be triggers for increased sFlt-1 production. A study of metformin showed it was able to prevent PE, reducing the production of sFlt-1 and sEng and ameliorating endothelial dysfunction through effects on mitochondria [202]. The protein statin exerts a protective effect on endothelial cells through induction of Hmox-1 expression and inhibition of sFlt-1 release, along with its antioxidant properties [203].

Vitamin D has a therapeutic effect on PE, as observed in the l-nitro-arginine methyl ester-induced PE rat models. Vitamin D supplementation was found to increase VEGF levels and decrease sFlt-1 and TNF-α levels in PE rats. Vitamin D also reduced oxidative stress by lowering the levels of malondialdehyde (plasma oxidative stress marker) [204]. Moreover, molecular hydrogen (H_2_) has therapeutic effects in several oxidative stress-related disorders. In the RUPP rat model of PE, H2 reduced mean arterial pressure and sFlt-1 expression. H2 also reduced sFlt-1 expression in villous explants taken from preeclamptic women. These studies show the preventive and therapeutic effects of H_2_ on PE [205].

A study with a CD-1 mouse model of PE showed maternal treatment with pravastatin prevents alterations in fetal brain development, growth, and metabolic functions [206,207]. It has been shown that PE alters brain development in sex-specific patterns, and prenatal pravastatin therapy prevents changes in neuroanatomic programming that occur in the preeclamptic CD-1 mouse model [206]. Pravastatin may exert its effects through pleiotropic mechanisms involving the pro-survival/antiapoptotic MAPK pathway in the placenta [208]. One more drug, edaravone (free radical scavenger), has inhibitory effects on the expression of sFlt-1 in the hypoxia-induced HTR8/SVneo trophoblast cell line. This compound showed a protective effect on the vascular development of human umbilical vein endothelial cells (HUVECs) in hypoxia, proving it to be a potential therapeutic agent for PE treatment [209]. Experimental animals administered exogenous VEGF121 also show an alleviation of PE symptoms [47].

## 5. Targeted Drug Delivery to the Placenta

There are currently no truly effective therapies and that drugs that could be useful to treat the placenta are often toxic systemically [210,211,212]. A placenta-specific drug delivery system has been developed using synthetic placental chondroitin sulfate A-binding peptide (CSA-BP). This peptide is derived from the malaria parasite *Plasmodium falciparum*, which mediates the binding of parasite-infected erythrocytes to the specific type of CSA in placental trophoblast cells (syncytiotrophoblasts). The study by Zhang et al. (2018) used CSA-BP-conjugated lipid-polymer nanoparticles successfully, demonstrating high drug-carrying capacity and stability and efficient delivery to the placenta [212]. This breakthrough finding has opened the opportunity of efficient therapeutic strategies for pregnancy complications.

## 6. Regenerative Therapy in PE

It has been suggested that placenta-derived mesenchymal stem cells (MSCs) have tremendous potential to treat the ischemic condition in PE [213]. MSCs are adult stem cells having anti-inflammatory, immune regulatory, and repair potential, as observed in animal models of hypertension, heart disease, and lung injury [214]. The regenerative potential of MSCs isolated from the placenta and umbilical cord could be exploited for the treatment of hypertension in PE [215]. MSCs were found to ameliorate the damage due to IUGR, kidney damage, and impaired spiral artery remodeling [216]. Human pluripotent stem cells have been successfully differentiated into multi-nucleated syncytiotrophoblasts (produced hCG) and HLAG+ extravillous trophoblast-like cells in in vitro culture [217]. Further studies on regenerative therapy are needed to develop alternative robust approaches for PE management.

## 7. Future Perspectives

Although preliminary studies suggest that the extracorporeal removal of circulating sFlt1 in PE prolongs pregnancy and improves the maternal symptoms of PE [34], this strategy should be considered very carefully, as sFlt1 also plays an essential role in protecting the fetus [27,180]. Additionally, a recent study in mice also suggests that reducing PlGF levels has a protective effect against the development of preeclampsia [54]. Thus, more research is needed to establish the pathogenesis of PE and the targeting of sFlt1 as a therapeutic strategy for the treatment of PE. Targeted drug delivery and regenerative therapy have a tremendous potential to prevent and manage PE, and more in-depth studies are required in this direction.
